# Stem/Progenitor Cells and Their Therapeutic Application in Cardiovascular Disease

**DOI:** 10.3389/fcell.2018.00139

**Published:** 2018-10-18

**Authors:** Yuning Hou, Chunying Li

**Affiliations:** Center for Molecular and Translational Medicine, Georgia State University, Atlanta, GA, United States

**Keywords:** stem cells, progenitor cells, endothelial progenitor cells, cardiovascular disease, cell therapy

## Abstract

Cardiovascular disease is the leading cause of death in the world. The stem/progenitor cell-based therapy has emerged as a promising approach for the treatment of a variety of cardiovascular diseases including myocardial infarction, stroke, peripheral arterial disease, and diabetes. An increasing number of evidence has shown that stem/progenitor cell transplantation could replenish damaged cells, improve cardiac and vascular functions, and repair injured tissues in many pre-clinical studies and clinical trials. In this review, we have outlined the major types of stem/progenitor cells, and summarized the studies in applying these cells, especially endothelial stem/progenitor cells and their derivatives, in the treatment of cardiovascular disease. Here the strategies used to improve the stem/progenitor cell-based therapies in cardiovascular disease and the challenges with these therapies in clinical applications are also reviewed.

## Introduction

According to the recent study, cardiovascular diseases (CVDs) are highly prevalent globally and produce immense health and economic burdens in the United States and the world (Writing Group Members et al., 2016). The pathophysiological and physiological changes accompanied with vascular aging lead to compromised cardiovascular functions and elevated risks of CVDs including atherosclerosis, hypertension, and diabetes in elder population (El Assar et al., 2012). Peripheral arterial disease (PAD) and coronary heart disease including myocardial infarction (MI) account for most of all CVDs (Writing Group Members et al., 2016). Except for genetic defects, most CVDs can be attributed to unhealthy lifestyle factors such as high fat diet, high salt diet, and smoking (Writing Group Members et al., 2016).

With the advances in our understanding of the underlying mechanisms of CVDs, breakthrough has been achieved in diagnosis and intervention, such as percutaneous coronary intervention (PCI), coronary artery bypass grafting (CABG), and heart transplantation. However, to some degree, these approaches can only delay the heart failure (Trevelyan et al., 2005). This burden of disease has driven the investigation of stem/progenitor cell-based therapy for the CVDs. Experimental studies suggested that administration of endogenous stem/progenitor cells may contribute to functional regeneration of infarcted myocardium and repair damaged/injured endothelial cells (Xu, 2006). Over the past decades, stem or progenitor cell-based therapy has emerged as a promising approach for the treatment of various CVDs, such as MI, heart failure, and PAD (Wollert and Drexler, 2010). The efficacy of various stem/progenitor cells including endothelial progenitor cells (EPCs) (Leistner et al., 2011), hematopoietic stem cells (HSCs) (Perin et al., 2012), cardiac stem cells (CSCs) (Makkar et al., 2012), and bone-marrow derived mononuclear cells (MNCs) (Wollert et al., 2017) in treating CVDs has already been evaluated in clinical trials. The potential therapeutic applications of stem/progenitor cells, such as embryonic stem cells (ESCs) (Shiba et al., 2012) and mesenchymal stem cells (MSCs) (Min et al., 2002), have been investigated in experimental and preclinical studies. As endothelial dysfunction is one of the major problems for CVDs, there is an increasing interest and ongoing efforts to study EPCs and other stem/progenitor cell-derived endothelial cells as potential sources for cell therapy (Reed et al., 2013). Here we summarize the studies using endothelial stem/progenitor cells and their derivatives, or sometimes oversimplified as “EPCs,” in the treatment of CVDs.

## Cell Spectrum of Stem/Progenitor Cell Derived Endothelial Cells

### MSCs

Mesenchymal stem cells were originally identified and characterized by Friedenstein et al. (1976) in the 1970s. MSCs have been found in multiple organs throughout the body including bone marrow (BM), umbilical cord, placenta, dental pulp, and adipose tissue and their characteristics have been reviewed recently (Karantalis and Hare, 2015). MSCs derived from BM, adipose tissue, and umbilical cord have been widely used in preclinical and clinical trials. It has been reported that adipose-derived stromal/stem cells (ASCs) possess strong angiogenic potential and paracrine activities (Bura et al., 2014). Early phase clinical trials have shown that ASC transplantation has improved rest pain, ulcer surface, walking distance, pain-free walking time, and transcutaneous oxygen pressure in PAD patients (Lee et al., 2012). MSCs possess several advantages as one of the promising candidates for stem/progenitor cell-based therapy: First, MSCs are easy to isolate and expand; Second, MSCs can secrete growth factors or directly differentiate into vascular cells or myocytes to contribute to arteriogenesis and angiogenesis (Wingate et al., 2014); Third, MSCs hold an immunoregulatory capacity and immunosuppressive effect indicating their potential of autotransplantation (De Miguel et al., 2012). These advantages enable MSCs to improve the neovascularization and blood flow in PAD and MI related ischemic tissues (Iwase et al., 2005; Gnecchi et al., 2006). Though with limitations such as the low retention and survival of transplanted MSCs (Muller-Ehmsen et al., 2006), the cell pretreatment and genetic engineering approaches will provide a promising future for MSC based therapy (Li et al., 2007).

### iPSCs

Induced pluripotent stem cells (iPSCs), which exhibit pluripotent differentiation and self-renewal potential that are similar to that of ESCs, was originally reported by Takahashi et al. (2007). By introducing four essential transcription factors (Oct3/4, Sox2, c-Myc, and Klf4) into fibroblasts, Yamanaka and colleagues have reprogrammed the cells into pluripotent stem cells. Using this technique, they and others have shown that iPSCs can be differentiated into endothelial cells (Sivarapatna et al., 2015). Studies have shown that iPSC derived endothelial cells are capable of angiogenesis and reendothelialization to form vascular networks *in vitro* (Suzuki et al., 2012). Preclinical studies also showed the promising therapeutic potential of iPSCs (Gu et al., 2012). Although teratoma formation (Seminatore et al., 2010) and the potential of tumorigenicity of transplanted cells (Yamanaka, 2012) are challenges in the clinical applications of iPSCs, iPSCs generated via non-genetic based techniques (Rhee et al., 2011) will improve the safety to overcome those disadvantage. Because iPSCs can be derived from mature somatic cells, the cell source is easy to obtain. Furthermore, the source of iPSCs can be autologous, so there is no need for immunosuppression when delivery. These features make iPSCs an attractive cell source for regenerative medicine.

### AFSCs

Amniotic fluid derived stem cells (AFSCs) have been documented to be a special type of stem cells that possess a comprehensive multi-differentiation potential (Romani et al., 2015). Preclinical studies have shown that AFSCs can differentiate into vascular cell lineages to improve blood supply (Maraldi et al., 2013) or promote the regeneration of myocytes through their paracrine effects (Bollini et al., 2011). Besides, AFSCs also possess several advantages which make them a potential therapeutic approach. First, ASFCs are easy to be obtained from amniocentesis specimens which are used for prenatal genetic diagnosis. Second, the obtained ASFCs, which are c-Kit positive, can be readily expanded *ex vivo* with a doubling time of 36 h. Third, ASFCs can be differentiated into cell types including adipogenic, osteogenic, myogenic, endothelial, neuronal, and hepatic lineages (Romani et al., 2015). More importantly, it has been recently reported that AFCSs can induce immunosuppressive activities of regulatory T cells (Tregs) to promote allograft survival in animal models of allogeneic transplantation (Romani et al., 2015). With more extensive studies being conducted, detailed molecular mechanisms have been proposed. A most recent study has demonstrated that several properties of AFSCs including immunoregulatory functions, cell differentiation toward multiple lineages, and migratory potency are regulated by sphingosine-1-phosphate (S1P) (Romani et al., 2018).

### MNCs

Mononuclear cells, which can be isolated from BM and peripheral blood, are extensively studied in tissue engineering and regenerative medicine. They can be harvested from BM and peripheral blood by density gradient centrifugation with no need for *ex vivo* expansion. Moreover, MNCs are heterogenic which contain several types of stem/progenitor cells such as MSCs and EPCs. These cells are capable of differentiating into vascular and/or myocytes, or secrete growth factors improving the regeneration of injured tissues (Karantalis et al., 2012). These features allow quick autologous application after harvest, so MNCs are widely used as therapeutic cells in CVDs (Goumans et al., 2014). However, recent systemic review and meta-analysis of the clinical efficacy of MNC transplantation only reveal modest clinical benefit. For PAD, improvements could be achieved in wound healing, amputation-free survival, pain-free walking, resting pain, and ulcer healing, but administration of MNCs could not improve the primary end-point of limb amputation compared with placebo (Rigato et al., 2017; Qadura et al., 2018). Another recent meta-analysis consisting of 2037 patients with acute MI has shown that MNC therapy only modestly improved left ventricular ejection fraction (LVEF) and infarct size (de Jong et al., 2014). Despite the publication bias and possible lack of statistical power, several aspects during MNC administration could be improved to achieve better clinical results, for instance, refinement of cell delivery strategy to enhance cell survival and function. Recent progress made in the decelluarized scaffolds, which create the scaffolds enriched in structural extracellular matrix components that support cell attachment and infiltration *in vitro* and *in vivo* (Crapo et al., 2011), stimulates great interest. Moreover, current genomic sequencing and proteomic techniques could also be utilized to identify essential pathways to improve the survival and function of transplanted cells.

### CPCs

After the introduction of cardiac progenitor cells (CPCs), researchers began to determine the possibility of the experimental and clinical usage of CPCs as a potential therapeutic agent. CPCs are a group of heterogeneous cells residing in the cardiac tissue (Senyo et al., 2013). After the identification of CPCs, researchers have discovered different cardiac resident cellular pools in human or murine heart, showing a variety of stem cell markers, including c-Kit^+^, stem cell antigen-1^+^ (Sca-1^+^), Islet 1^+^ (Isl-1^+^), stage-specific embryonic antigen-1^+^ (SSEA-11^+^), cardiospheres (CS), cardiospheres-derived (CD), and side population (SP), which has recently been reviewed extensively by Bianconi et al. (2017, 2018). CPCs can self-renew, and they can also differentiate into three different cardiac cell types including cardiomyocytes, smooth muscle cells and endothelial cells (Sturzu and Wu, 2011; Bianconi et al., 2018). It has been reported that embryonic heart tubes derived CPCs can differentiate into pacemaker-like cells through endothelin-1 factor involved signaling (Zhang et al., 2012). Recently, engineered cardiac pacemakers containing both CPC-derived pacemaker-like cells and EPCs have demonstrated the promising potential to ameliorate sinus node malfunction (Zhang et al., 2017). Meanwhile, accumulating studies have shown that CPCs promote cardiac tissue restoration after CVD by releasing anti-apoptotic and angiogenic signals in a paracrine manner (Ibrahim et al., 2014). It has been shown that CPC-derived exosomes promoted angiogenesis, cardiomyocyte survival and proliferation, and reduced cell apoptosis (Marban, 2014). Analysis of CPC-based clinical trials has revealed that patients suffering from heart-related diseases benefit from CPC-based therapy (Bianconi et al., 2018).

### EPCs

Asahara et al. (1997) initially isolated angioblasts with endothelial lineage potential from human peripheral blood and named them “EPCs.” They also found that these EPCs can differentiate into endothelial-like cells *in vitro* and participate in neovascularization in animal models of ischemia. Later, EPCs have been shown to migrate to peripheral blood from BM to participate in repairing dysfunctional endothelia and decreasing cardiovascular risk factor related endothelial injury by directly infusing into and forming new vessels or secreting pro-angiogenic growth factors or cytokines (Asahara et al., 2011). Although an increasing number of reports have been documented to identify EPCs, there is still a lack of unambiguous and consistent definition of EPCs. Generally, EPCs are a group of cells which are characterized by positively expressing VEGFR2/Flk1, CD133/AC133, and CD34 at early stages; while at late stages when they gradually differentiate into endothelial cells, EPCs start to express endothelial markers including VE-cadherin, vWF, and endothelial nitric oxide synthase (eNOS) (Ambasta et al., 2017). Accumulating studies indicates that early EPCs promotes angiogenesis in a paracrine manner, and the late stage EPCs directly participate in endothelial neovascularization (Ambasta et al., 2017).

## EPC Based Cell Therapy in CVDs

It is well-known that the integrity and functional activity of the endothelial monolayer are maintained by replication and migration of neighboring mature endothelial cells under physiological conditions. However, a series of clinical and pre-clinical studies have provided the evidence that in conditions of endothelial injury, regeneration of endothelial monolayer is assisted by EPCs homing to the artery wall. A critical early event in CVDs is endothelial dysfunction, which is perpetuated during the exposure of cardiovascular risk factors including hypercholesterolemia, metabolic syndrome, diabetes, hypertension, dyslipidemia, aging, and smoking. It has been reported that the number of circulating EPCs is inversely correlated with the presence of cardiovascular risk factors (Mannarino and Pirro, 2008; Pirro et al., 2015). Over the last two decades, extensive investigations in clinical and preclinical studies indicate that EPCs are a promising option to treat CVDs such as MI. The findings of EPC cell therapy for MI in animal studies have been summarized in **Table 1**. Accumulating clinical trials have evaluated the safety and efficacy of EPCs for CVDs treatment, as summarized in **Table 2**. As revealed in **Table 2**, the clinical outcomes of the stem/progenitor cell-based therapy only achieved modest benefits, so more strategies should be employed to improve the stem/progenitor cell-based therapy.

**Table 1:** Stem cell/EPC therapy in animal models of MI.

Animal model	Transplanted cell type	Delivery strategy	Outcomes	Reference
Mouse MI	Mouse BM-EPCs	Intravenous injection	EPC incorporated into neovascularization foci at infarct border	Asahara et al., 1999
Mouse MI	Bone marrow derived mouse Lin- c-kit+	Intramyocardial injection	Newly formed myocardium occupied 68% of the infarcted portion of the ventricle were observed	Orlic et al., 2003
Rat MI	Human peripheral blood EPCs	Intravenous injection	EPCs incorporated into foci of neovascularization, smaller ventricular dimensions and ventricular scarring; increased fractional shortening, capillary density	Kawamoto et al., 2001
Rat MI	Human peripheral blood CD34^+^ cells	Tail vein injection	Decreased apoptosis of hypertrophied myocytes in the peri-infarct region, reduced collagen deposition, increased myocardium survival and cardiac function	Kocher et al., 2001
Pig MI	Pig MNCs	Trans-endocardial injection	Increased systolic function, regional blood flow, collateral vessel formation, and decreased ischemic area	Kamihata et al., 2002
Pig MI	Pig MSCs	Intramyocardial injection	Decreased degree of contractile dysfunction and wall thinning	Shake et al., 2002
Rat MI	Rat MSCs transduced Akt1	Intramyocardial injection	Inhibited the process of cardiac remodeling, restored myocardial volume	Mangi et al., 2003
Rat MI	Human peripheral blood CD34^+^ angioblasts (EPCs)	Tail vein injection	Dose-dependent neovascularization with development of larger-sized capillaries; improve cardiac function through inhibiting apoptosis and promoting proliferation of cardiomyocytes	Schuster et al., 2004
Rat MI	Rat ASCs	Sheet technology (monolayered cell graft placed on the surface of the anterior scar)	ASCs reversed wall thinning in scar area and improve cardiac function. ASCs triggers angiogenesis and differentiate into vessels and cardiomyocytes	Miyahara et al., 2006
Rat MI	Rat umbilical cord blood CD133^+^ cells	Intravenous infusion	Scar thinning and LV systolic dilatation were prevented	Leor et al., 2006
Pig MI	Pig CD34^+^	Intracoronary injection	Improved cardiac repair and collateral vessel formation	Zhang et al., 2007
Rat MI	Human EPCs accompanied with SDF-1	Intramyocardial injection	Improved fractional shorting, left ventricular developing pressure, coronary flow rates, and neovascularization. Reduced the number of inflammatory cells and the rate of apoptotic cells	Schuh et al., 2008
Mouse MI	Human myoendothelial cells	Intramyocardial injection	Improved left ventricular function. Increased angiogenesis. Stimulated proliferation and survival cardiomyocytes. Reduced scar tissue	Okada et al., 2008
Rat MI	ECM scaffold supplemented with EPCs primed with SDF-1	Sutured to the anterolateral left ventricular wall	Increased VEGF level, vessel density, microvascular perfusion, vasculogenic response, and decreased scar formation	Frederick et al., 2010
Rat MI	Rat peripheral blood EPCs transduced with IGF-1	Intramyocardial injection	Increased cardiac function, cardiomyocyte proliferation, and capillary density, decreased cardiac apoptosis	Sen et al., 2010
Pig MI	Human embryonic stem cells	Fibrin-cell path applied to the LV anterior wall of the MI area	Improved left ventricular function and neovascularization	Xiong et al., 2011


**Table 2:** Stem/progenitor cell/EPC therapy in clinical studies of CVDs.

Trial design	Disease	Cell type	Delivery strategy	Outcomes	Reference
22 Bilateral ischemia patients, 25 unilateral ischemia patients, within-patient controls	CLI	MNCs derived from BM or peripheral blood (PB)	Intramuscular injection	Improved transcutaneous oxygen pressure (TcPO2), rest pain, pain-free walking time, and ankle-brachial index (ABI)	Tateishi-Yuyama et al., 2002
7 Patients, no controls	CLI	BM derived MNCs	Intramuscular injection	Improved ABI, TcPO2, pain-free walking time, and leg blood flow	Higashi et al., 2004
6 Patients, no controls	Acute myocardial infarction (AMI)	PB CD34^+^ cells	Intracoronary injection	Improved wall motion score index	Blocklet et al., 2006
44 Cell-injected patients, 22 control	AMI	BM-MNCs	Intracoronary injection	Increased LVEF and peak systolic velocities the infarcted wall longitudinal contraction	Meluzin et al., 2006
41 Cell-injected patients, 45 control	ST-segment elevation MI	BM-MNCs	Intracoronary injection	Increased LVEF, no improvement of myocardial viability of infarcted area	Cao et al., 2009
7 Patients, non-randomized control	Anterior MI	PB CD34^+^ cells	Transcoronary, intracoronary infusion	Decreased end-systolic volume	Dedobbeleer et al., 2009
7 Patients, no controls	AMI	PB CD34^+^ cells	Intracardiac infusion	Increased LVEF, vascularization, and the regeneration of myocardial structure	Pasquet et al., 2009
28 Patients, no controls	CLI	CD34^+^ CD133^+^ EPCs	Intramuscular injection	Improved limb salvage rate and attenuated pain scale	Lara-Hernandez et al., 2010
25 Cell-injected patients, 25 placebo-injected patients; Randomized double-blinded trial	Chronic myocardial ischemia	BM-MNCs	Intramyocardial infusion	Improved E/e’ and E/A ratios, increased LVEF	van Ramshorst et al., 2011
112 Cell-injected patients, 56 placebo-injected patients; Phase II, prospective, double-blinded, randomized trial	Refractory angina	CD34^+^ cells	Intramyocardial infusion	Improved exercise tolerance	Losordo et al., 2011
71 Cell-injected patients, 71 placebo-injected patients; Phase III, randomized, double-blinded trial	MI	CD133^+^ cells	Intramyocardial infusion	Patients received CD133^+^ cell injection had higher LVEF	Donndorf et al., 2012
17 Patients, no control, Phase I/II clinical trial	CLI	Granulocyte-colony stimulating factor (GCSF) mobilized CD34^+^ cells	Intramuscular injection	Improved toe brachial pressure index and TcPO2, pain scale, ulcer size, and exercise tolerance	Kinoshita et al., 2012
25 Patients, no control	CLI	GCSF mobilized PB CD34^+^ cells	Intramuscular injection	Improved pain-free walking time, ABI, TcPO2, and decreased pain score	Dong et al., 2013
11 Patients, no control; Phase II clinical trial	CLI	GCSF mobilized PB CD34^+^ cells	Intramuscular injection	Increased pain scale, skin perfusion pressure, TcPO2, total walking distance, toe brachial pressure index, and CLI-free ratio	Fujita et al., 2014
49 Patients, no control	CLI	BM-MNCs	Intramuscular and intraarterial injection	Limb amputations were delayed; Improved ABI, rest pain, and ulcer healing	Franz et al., 2015


## EPC Based Therapy for Ischemic Vascular Diseases

The therapeutic efficacy of EPCs was not only documented in the studies of CVDs but also in the peripheral artery diseases (PAD). PAD is commonly referred to as the ischemia of limbs associated with atherosclerotic occlusion (Ouriel, 2001). Peripheral arteries supply oxygenated blood and nutrients to the legs and feet and narrowing of these arteries results in PAD. The most common symptom of PAD is the pain with walking which is also known as intermittent claudication (Ouriel, 2001). Critical limb ischemia (CLI) is the most severe clinical manifestation of PAD affecting a limb, if not interrupted, CLI could lead to ischemic ulcerations or even gangrene (Ouriel, 2001). In preclinical studies, the most adopted animal model is the hindlimb ischemia model (HLI) (Niiyama et al., 2009). In the HLI model, the femoral artery is ligated to reduce the blood supply to the lower leg which induces the angiogenesis to compensate for the reduced blood flow (Limbourg et al., 2009). The therapeutic efficacy of EPCs have been evaluated by this model by many groups, and **Table 3** summarizes the preclinical animal studies of EPC cell therapy for PAD.

**Table 3:** Stem cell/EPC therapy in animal studies of PAD.

Animal model	Transplanted cell type	Delivery strategy	Outcomes	Reference
Mouse and rabbit HLI	Human CD34^+^; mouse Flk-1^+^	Tail vein injection	EPC incorporated into sites of active angiogenesis	Asahara et al., 1997
Mouse HLI	Human EPC	Intracardiac injection	Ischemic hindlimb blood flow increased, capillary density increased, limb loss rate decreased	Kalka et al., 2000
Rat HLI	Human CD34^+^ NMC (EPCs)	Intramuscular injection	Neovascularization and blood flow increased in ischemic hindlimb	Murohara et al., 2000
Mouse HLI	Human CD34^+^ cells	Intramuscular injection	Blood flow restored in diabetic mice but not in non-diabetic mice	Schatteman et al., 2000
Rabbit HLI	Rabbit BM-MNCs	Intramuscular injection	More angiographically detectable collateral vessel, improved blood perfusion	Shintani et al., 2001
Mouse HLI	VEGF gene transduced Human EPCs	Tail vein injection	Neovascularization and blood flow recovery improved, and limb necrosis was reduced	Iwaguro et al., 2002
Mouse HLI	Human EPCs accompanied with SDF-1	Intramuscular SDF-1 and intravenous EPC injection	Improved local accumulation of EPCs in ischemic muscle, ischemic tissue perfusion, and capillary density	Yamaguchi et al., 2003
Mouse HLI	Human cord blood CD34^+^ KDR^+^ or CD34^+^ KDR-cells	Intramuscular injection	CD34^+^ KDR^+^ cells significantly improved limb salvage and neovascularization, reduced endothelial cell apoptosis and interstitial fibrosis compared with CD34^+^ KDR-cells	Madeddu et al., 2004
Mouse HLI	Human umbilical cord blood CD133^+^ EPCs	Tail vein injection	Increased neovascularization and improved ischemic limb salvage	Yang et al., 2004
Rat HLI	Human peripheral blood CD133^+^ progenitor cells	Intramuscular injection	Increased arteriole and capillary density	Suuronen et al., 2006
Mouse HLI	Human EPCs and smooth muscle progenitor cells	Intravenous injection	Vessel density and foot perfusion increased	Foubert et al., 2008
Mouse HLI	Mouse MNCs	Intramuscular injection	Increased blood flow ratio and capillary density; improved ankle-brachial index value, walking distance, pain scale, and TcPO2	Zhang et al., 2008
Mouse HLI	Human iPSC-ECS	Intramuscular injection	Increased capillary density and blood perfusion ratio	Rufaihah et al., 2011
Mouse HLI	Human HUVECs and umbilical cord MSCs	Intramuscular injection	Blood perfusion recovered, increased vessel formation	Chen et al., 2013
Mouse HLI	Human MNCs, ESC, and iPSC	Intramuscular injection	Increased neovascularization and decreased hindlimb ischemia	Lai et al., 2013
Mouse HLI	Human AFSCs	Intramuscular injection	Increased limb salvage, limb blood perfusion, and capillary and arteriole density	Liu et al., 2013


## Approaches for Enhancing EPC Therapy in Diseases

Although EPCs possess exciting therapeutic potency, their limited plasticity and amount in patients with ischemic cardiac or ischemic vascular disease have become the obstacle to the success in EPC therapy. It has been reported that compromised EPC availability and repair potential to regenerate the injured endothelial monolayer mainly resulted from the influence of cardiovascular risk factors such as aging, smoking, diabetes, hypertension, and hypercholesterolemia (Pirro et al., 2008, 2012). As summarized, the clinical outcome of EPC based therapy was modest, and large-scale clinical trials have not been conducted. One of the reasons is that there is a lack of suitable transplantation models. Studies in animal models suggested that BM-MNCs or EPCs could home to ischemic tissues and restore the blood supply, however, during atherosclerosis acute surgical resection has little resemblance to chronic occlusion (Qadura et al., 2018). Moreover, because of the heterogeneity between patients, in clinical trials, the selection of patient population for stem/progenitor cell-based therapy may not be optimized. These problems should be addressed before the clinical transfer of EPC based cell therapy. Therefore, an increasing number of studies have been focusing on the strategies to enhance the therapeutic efficacy of EPCs (Penn and Mangi, 2008). Various modifiers including chemokine receptors, growth factors, signaling molecules or factors, medicines, and physical exercise have been demonstrated to enhance the therapeutic effects of EPCs.

The key factors shown to enhance the cell-based therapeutics in CVDs include but not limited to: chemokine receptors such as CXCR2 (Hou et al., 2015), CXCR4 (Jujo et al., 2013), CX3CR1 (Herlea-Pana et al., 2015), CXCR7 (Zhang et al., 2014), and CCR5 (Zhang et al., 2015); growth factors and their receptors such as VEGF1/2/3 (Shintani et al., 2006; Smadja et al., 2007), PDGF (Rosell et al., 2013), FGF-1/2 (Rosell et al., 2013; Chien et al., 2016), and so on. Signaling molecules and factors such as eNOS/nitric oxide (Kaur et al., 2009; Cui et al., 2011), AMP-activated protein kinase (AMPK) (Wang X.R. et al., 2011), heme-oxygenase-1 (HO-1) (Sambuceti et al., 2009), and manganese superoxide dismutase (MnSOD) (Marrotte et al., 2010), have also been shown to play important roles in EPC biology. Additionally, several transcription factors signaling including Homeobox A9 (HOXA9), Akt/Forkhead box-containing protein O subfamily (Akt-FOXO), and peroxisome proliferator-activated regulator-gamma (PPARγ) have been suggested to be involved in regulating the function of EPCs (Pirro et al., 2008). Moreover, many medications used for prevention of CVDs have also been shown to increase the level of EPCs such as statins (Pirro et al., 2009; Wang W. et al., 2011) and angiotensin II receptor antagonists (Pelliccia et al., 2010), etc. Wang W. et al. (2011) demonstrated that statins could improve the mobilization, derivation, and colonial growth of late outgrowth EPCs. They have also shown that pravastatin increased the capillary density in chronic myocardial ischemia by 46% in an animal model. More interestingly, it has been reported that exercise could improve the function of EPCs (Guo et al., 2017). The underlying mechanism between exercise and EPCs has mainly been linked to CXCR4 signaling, VEGF release, and nitric oxide (NO) bioavailability. VEGF has been shown to be important in angiogenesis. Studies have shown that after exercise training, the expression level of VEGF and its receptors in mice were significantly increased in post-MI. Meanwhile, the mice preconditioned with exercise expressed higher level of VEGF and its receptors compared with mice without exercise preconditioning (Wu et al., 2009). In an animal model of hypertension, exercise significantly increased EPC levels and also resulted in vascular repair in a VEGF/eNOS dependent manner (Fernandes et al., 2012). Studies also demonstrated that exercise training increased the expression level of CXCR4 and phosphorylation level of Janus kinase-2 (JAK-2) of EPCs, improved the endothelial function *in vitro* and reendothelialization capacity of EPCs *in vivo*(Xia et al., 2012).

Studies have been performed to identify the key regulators to rescue the defective functions of EPCs from patients exposed to cardiovascular risk factors such as diabetes and aging. For instance, EPC transduced with Akt/HO-1 displayed increased MI recovery in nude mice (Brunt et al., 2012). The importance of eNOS in EPC angiogenesis has also been evaluated by different groups. In a rat balloon injury model, the neointimal hyperplasia was inhibited, and vascular function was restored by transplanting eNOS overexpressed EPCs (Cui et al., 2011). Also, it has been shown that overexpressing eNOS in EPCs isolated from coronary artery disease displayed increased functions such as proliferation, differentiation, migration, and integration into tube-like structures *in vitro* (Kaur et al., 2009). Accumulating studies have demonstrated the importance of chemokine receptors and their cognate ligands in EPC survival and function. CXCR4 has been shown to play a critical role in EPC mobilization and angiogenesis *in vivo* (Jujo et al., 2013). Recently, we have shown that CXCR2 macromolecular signaling complex is essential in mediating EPC homing and angiogenesis *in vitro* and *in vivo* (Hou et al., 2015). Therefore, approaches such as genetic modification of EPCs to modify the expression of the chemokine receptors and growth factor receptors, or pretreatment of cells with chemokines or growth factors to improve the angiogenic signaling activities, rejuvenate the cells, or enhance the survival of EPCs could be investigated to address the limitations of EPC transplantation. Moreover, the findings of the positive effects of medications and physical exercise provide additional options to enhance the efficacy of EPC therapy in cost-efficient manner.

## Conclusion

Although clinical trials and preclinical studies have shown that EPCs and other stem cell and progenitor cells based therapy possess great therapeutic potential to improve cardiac function and blood perfusion in MI and PAD, obstacles still exist to be overcome before widespread application of EPCs in the treatment of CVD (Roediger, 1980). Cell isolation, characterization, modification, and processing strategies must be further studied and refined to achieve enhanced therapeutic efficacy. For instance, there is still lack of consistent definition of EPCs, so further study is needed to standardize methods to define EPCs, through both lineage tracing and functional analysis (Masuda et al., 2011). Meanwhile, upregulation of certain circulating progenitor cells such as circulating osteoprogenitor cells may result in vascular calcification which is a cardiovascular risk factor (Pirro et al., 2013). Moreover, the cell infusion approach, dosing regimens, as well as the cell survival after delivery are also needed to be improved to achieve optimal outcomes (Freyman et al., 2006). Due to the possibility of occurrence of teratoma formation and tumorigenesis, especially during the transplantation of iPSCs, the safety of the stem/progenitor cell-based therapies should also be monitored (Yamanaka, 2012). In summary, previous clinical trials and preclinical studies have shed light on the EPC based therapy for treating CVDs. With more efforts to understand the biology of stem/progenitor cells and continued commitment to preclinical and clinical studies, stem/progenitor cell-based therapy may present an integral part of routine regenerative therapy for CVDs in the future.

## Author Contributions

YH wrote the manuscript and participated in edits. CL revised and edited the entire manuscript.

## Conflict of Interest Statement

The authors declare that the research was conducted in the absence of any commercial or financial relationships that could be construed as a potential conflict of interest.
